# Impacts of COVID-19 on US agri-food supply chain businesses: Regional survey results

**DOI:** 10.1371/journal.pone.0281930

**Published:** 2023-02-22

**Authors:** Hikaru Hanawa Peterson, Gigi DiGiacomo, Christa D. Court, Michelle Miller, Gustavo Oliveira, Andrew W. Stevens, Li Zhang, Lauri M. Baker, Joseph Nowak, Eyrika Orlando, Bijeta Bijen Saha

**Affiliations:** 1 Department of Applied Economics, University of Minnesota–Twin Cities, Saint Paul, Minnesota, United States of America; 2 Food and Resource Economics Department, University of Florida, Gainesville, Florida, United States of America; 3 Center for Integrated Agricultural Systems, University of Wisconsin–Madison, Madison, Wisconsin, United States of America; 4 Graduate School of Geography, Clark University, Worcester, Massachusetts, United States of America; 5 Department of Agricultural and Applied Economics, University of Wisconsin–Madison, Madison, Wisconsin, United States of America; 6 Department of Anthropology and Sociology, Department of Environmental Studies, Amherst College, Amherst, Massachusetts, United States of America; 7 Department of Agricultural Education and Communication, University of Florida, Gainesville, Florida, United States of America; 8 Department of Forest Resources, University of Minnesota–Twin Cities, Saint Paul, Minnesota, United States of America; University of Hail, SAUDI ARABIA

## Abstract

Visible disruptions of appropriate food distribution for end consumers during the onset of the COVID-19 pandemic prompted calls for an urgent, renewed look at how the U.S. agri-food system is impacted by and responds to pandemics, natural disasters, and human-made crises. Previous studies suggest the COVID-19 pandemic yielded uneven impacts across agri-food supply chain segments and regions. For a rigorously comparable assessment of the impact of COVID-19 on agri-food businesses, a survey was administered from February to April 2021 to five segments of the agri-food supply chain in three study regions (California, Florida, and the two-state region of Minnesota-Wisconsin). Results (N = 870) measuring the self-reported changes in quarterly business revenue in 2020 compared to businesses’ typical experience pre-COVID-19 suggest significant differences across supply chain segments and regions. In the Minnesota-Wisconsin region, restaurants took the largest hit and the upstream supply chains were relatively unaffected. In California, however, the negative impacts were felt throughout the supply chain. Two factors likely contributed to regional differences: (1) regional disparities in pandemic evolution and governance and (2) structural differences in regional agri-food systems. Regionalized and localized planning and the development of best-practices will be necessary for the U.S. agri-food system to enhance preparedness for and resilience to future pandemics, natural disasters, and human-made crises.

## Introduction

The functions of a food system are to provide adequate nourishment to the populace and to support the livelihood of people who supply food [1]. Supply chains are business-to-business relationships that connect producers, processors, distributors, and retailers to consumers and are key to a functioning food system. The COVID-19 pandemic caused major disruptions to the agri-food supply chain exposing acute structural vulnerabilities and undermining immediate food security worldwide [2]. In North America, studies have noted disruptions in labor supply, particularly severe for meat processing, fruit and vegetable production, and food services [3, 4]. Across different geographics, revenue in the food services sector declined drastically during the first and second quarters of 2020, while revenue in the retail sector changed little [5, 6].

Studies examining national data in 2020 have largely concluded that the impact of COVID-19 was short-lived, citing the resilience of the overall North American food systems [6, 7]. Several studies have suggested the impact of COVID-19 on food supply chains differed across sub-sectors [8, 9] depending on the market structure of the industry [10], space, and time [11–13]. Rigorous estimates of the associated financial losses by region and supply chain segment are essential for future disaster preparedness planning [14]. Yet, most existing studies have focused on select supply chain segments (e.g., restaurants) or agricultural commodity sectors (e.g., livestock or produce) [7, 9, 11, 15–18].

This study fills this critical void in the literature by reporting comparable estimates of the impact across agri-food supply chain segments and commodity sectors. We collected data using a harmonized survey instrument across multiple US regions and supply chain segments to test the hypothesis that the impact of the COVID-19 pandemic on agri-food supply chain operations differed across supply chain segments and regions. Our approach allows for a more comprehensive, comparative analysis of regional food systems.

## Materials and methods

California, Florida, and the two-state region of Minnesota-Wisconsin were chosen as the study regions based on the established networks of the project team. The three study regions highlight regional differences in sociodemographic characteristics, COVID-19 prevalence, and public health measures implemented, as well as the size, scope, and structure of the regional agri-food system. Agri-food supply chain segments—including input supply, farming, processing, distribution/wholesaling, retailing, and waste/recovery—account for 17% to 23% of state-wide employment in the study states (Table 1). Sales from the six agri-food supply chain segments constitute 22% and 25% of the state gross domestic product in California and Florida, respectively, compared to 38–39% in Minnesota and Wisconsin. Agricultural production in the three regions, where climate and growing conditions vary dramatically, is diverse. Top grossing commodity groups in California and Florida are fruits, vegetables, and tree nuts, with roughly a quarter of agricultural sales value coming from livestock. The primary crops in terms of value for Minnesota and Wisconsin are grains and oilseeds, with livestock accounting for 45% and 64% of agricultural sales value, respectively.

**Table 1:** Characteristics of the regional agri-food system, by study region^a^.

			California		Florida		Minnesota		Wisconsin	
2017 total employment in food system^b^ (%state employment)			19%		17%		21%		23%	
	Number of establishments by sector (%total food system)									
		Input supply		1%		1%		1%		1%
		Farming		38%		45%		79%		75%
		Processing		5%		3%		3%		3%
		Distribution & Wholesaling		4%		3%		1%		1%
		Retailing		51%		47%		16%		20%
		Waste & Recovery		1%		1%		1%		0%
										
2017 total sales of the food system (%state GDP)^c^			22%		25%		39%		38%	
	Sales by sector (% total food system sales):									
		Input supply		5%		5%		14%		8%
		Farming		7%		3%		13%		9%
		Processing		22%		13%		33%		45%
		Distribution & Wholesaling		27%		31%		18%		14%
		Retailing		37%		47%		21%		23%
		Waste & Recovery		2%		2%		1%		1%
2017 value of agricultural sales ($ million)			38,195		6,233		15,560		9,666	
	Percent livestock and products		26%		23%		45%		64%	
	Percent vegetables, fruits & tree nuts		57%		35%		2%		6%	
	Percent grains, oilseeds & other crops		17%		42%		53%		29%	

^a^Data available from [27] unless noted otherwise.

^b^Food system includes agri-food supply chain sectors of input supply, farming, processing, distribution and wholesaling, retailing, and waste and recovery [28].

^c^Calculated from 2017 Economic Census [29] following [28].

A validated agricultural disaster assessment instrument [19] was adapted to survey five agri-food supply chain segments: agricultural production, food processing/manufacturing, grocery wholesaling, food and beverage retailing, and restaurants. The survey was fielded from February 1, 2021, to April 15, 2021 using the online platform Qualtrics. The survey questionnaire, ranged from 21–154 questions depending on business status (closed, temporarily closed, open) and the supply chain segment(s) represented, was distributed to businesses in the study regions.

Survey distribution lists were compiled from Data Axel/Reference Solutions and from private and non-profit membership organizations representing all segments of the agri-food supply chain. Postcards with a QR code linking to the survey were distributed by mail to businesses representing the middle segments of the supply chain to improve the response rate from these under-studied groups. The respondents in Minnesota-Wisconsin were offered an opportunity to opt into a gift card drawing of $200, and the respondents in California were entered into a drawing for $200 e-gift certificates. Incentives were not offered in Florida based on familiarity with and success of historical post-disaster communications from the University of Florida. Follow-up reminders were issued every two weeks throughout the survey period. The study participants were informed of the study protocol in the cover page of the survey. They were asked to proceed with the survey questions only if they consented to the study protocol. The study was reviewed and approved by the Institutional Review Boards at authors’ respective institutions.

The responding businesses were first asked how business sales revenues were split across the five supply chain segments in a typical year prior to the pandemic. The key questions of interest were a series asking responding businesses to estimate how their food-related sales revenue changed for each quarter of 2020 compared to the same quarter in an average year. Thus, respondents reported their business’s estimated changes in food-related sales revenue for the supply chain segments in which they were involved. The responses were separated by quarters, supply chain segments, and study regions, and were respectively tested for equivalence to no change in sales revenue, using the non-parametric sign test.

We also tested for pair-wise equivalence of responses between quarters, supply chain segments, and study regions, using the Wilcoxon rank-sum test. Specifically, for the change in revenue in 2020 from their previously typical levels in segment *g*, quarter *i*, and region *j* (*γ*_*g*,*i*,*j*_), we tested the following hypotheses:

$$\gamma_{g,i,j}=\gamma_{g\prime \neq g,i,j}$$ (1)

$$\gamma_{g,i,j}=\gamma_{g,i\prime \neq i,j}$$ (2)

$$\gamma_{g,i,j}=\gamma_{g,i,j\prime \neq j}$$ (3)

for *g*, *g*′ = {production agriculture, food processing/manufacturing, grocery wholesale, food and beverage retail, restaurants}, *i*, *i*′ = 1, …, 4, and *j*, *j*′ = {California, Florida, Minnesota-Wisconsin}. Eq 1 examines the differences in change in revenue across supply chain segments in each region and quarter; Eq 2 tests for differences in changes across quarters for each supply chain segment and region; and Eq 3 looks at the regional differences. There are 120, 90, and 60 pairs in each equation for a total of 270 pairwise test.

## Results

Survey responses (N = 870) were collected from study regions in the following proportions: California (50%), Florida (11%), and Minnesota-Wisconsin (38%). Respondents represented all five segments of the agri-food supply chain with the largest number involved in restaurants (33%), followed by production agriculture (22%), food and beverage retailing (21%), food processing/manufacturing (8%), grocery wholesaling (7%), and other food-related entities such as food banks and institutional food services (9%). Note that respondents could indicate their involvement in more than one supply chain segment. The segment representation in the sample is comparable to the numbers of establishments according to the 2017 Economic Census in the study states (Table 1).

Table 2 summarizes characteristics of the businesses in our sample. Responding businesses from California and Minnesota-Wisconsin were relatively similar in terms of both 2019 annual sales revenue and total employment. Their median sales revenues were around half a million dollars, while sales revenues reported by Florida businesses were higher with a median of $2 million. The median numbers of employees in California and Minnesota-Wisconsin were around a dozen, compared to 20 among Florida respondents. Three quarters of the responding businesses remained open throughout the pandemic, with regional proportions ranging from 69% in California to 97% in Florida.

**Table 2:** Descriptive summary of survey respondents, by study region.

			California		Florida		Minnesota-Wisconsin	
*Number of respondents* (*% total*)			439	(50%)	97	(11%)	334	(38%)
2019 sales revenue, *N* (*% total*)			308	(53%)	51	(9%)	226	(39%)
	*Mean* (*Std*. *Dev*.), $1,000		11,799	(131,692)	17,486	(46,827)	18,121	(122,099)
	*Median*, $1,000		585		2,000		350	
	*5th percentile*, $1,000		8		0		2	
	*95th percentile*, $1,000		19,797		90,000		25,000	
2019 total employment, *N* (*% total*)			311	(52%)	51	(9%)	234	(39%)
	*Mean* (*Std*. *Dev*.), # employees		54	(188)	55	(101)	81	(659)
	*Median*, # employees		12		20		10	
	*5th percentile*, # employees		0		0		0	
	*95th percentile*, # employees		150		220		150	
Business status, *N (% total)*			214	(50%)	33	(8%)	178	(42%)
	Business remained open throughout		148	(69%)	32	(97%)	144	(80%)
		pandemic, *N* (*% state*)						
	Business closed temporarily during the pandemic		37	(17%)	1	(3%)	14	(8%)
		but is currently open, *N* (*% state*)						
	Business closed as a result of the pandemic, *N*		29	(14%)	0	(0%)	21	(12%)
		(*% state*)						

Table 3 presents regional differences in the types of shocks experienced by businesses during the first and second halves of 2020. More than 80% of respondents experienced some changes related to inputs, sales revenue, and finances in both halves of 2020, with fewer respondents reporting experiences in the second half. Specifically, greater proportions of California respondents indicated a shortage of service providers or loss of existing suppliers and buyers in both halves of the year, suggesting that struggles in one part of the supply chain spread to businesses in the adjacent segments, potentially metastasizing challenges across the entire supply chain.

**Table 3:** Reported experiences during 2020.

	During Jan-June of 2020				During July-Dec of 2020			
	All	CA	FL	MN-WI	All	CA	FL	MN-WI
Number of responses	351	175	31	145	333	165	29	139
	------------------------------*Percent of number of responses*------------------------------							
***Inputs***	88%	89%	81%	90%	84%	82%	72%	88%
Shortage of materials	45%	43%	35%	49%	35%	35%	24%	37%
Shortage of service providers	26%	30%	13%	23%	21%	26%	14%	16%
Shortage of packaging supplies	40%	41%	32%	39%	32%	32%	17%	37%
Change in costs of inputs/products	64%	62%	68%	66%	56%	56%	24%	63%
Loss of existing suppliers	19%	24%	16%	13%	16%	22%	7%	11%
Approached by new suppliers	14%	14%	16%	14%	14%	15%	3%	14%
Changes in supplier delivery formats	32%	34%	32%	30%	26%	25%	34%	24%
Suppliers unable to meet contract obligations	19%	20%	26%	17%	14%	16%	7%	14%
***Sales***	93%	96%	87%	90%	89%	93%	69%	89%
Loss of existing buyers/customers	71%	81%	71%	59%	64%	71%	45%	59%
Approached by new buyers/customers	31%	30%	23%	34%	29%	28%	10%	35%
New packaging/delivery requirements	34%	38%	29%	30%	28%	32%	7%	29%
Change in price of products sold/shipped	46%	45%	42%	48%	43%	42%	34%	45%
***Finances***	91%	95%	90%	87%	89%	95%	79%	85%
Increase in cashflow	20%	14%	16%	29%	24%	16%	31%	32%
Decrease in cashflow	70%	81%	68%	57%	65%	78%	48%	53%
Difficulty meeting contractual obligations	20%	25%	23%	13%	14%	18%	10%	10%
***Labor***	60%	61%	39%	63%	56%	59%	38%	57%
Labor shortage: sickness	34%	38%	16%	34%	38%	42%	24%	37%
Labor shortage: immigration restrictions	4%	5%	6%	3%	6%	7%	10%	3%
Labor shortage: strikes/other labor disputes	1%	2%	0%	0%	3%	4%	0%	1%
Labor shortage: day-care/school closures	27%	25%	26%	31%	26%	28%	14%	25%
Labor shortage: health measure compliance	32%	34%	19%	32%	0%	0%	0%	0%
Loss of volunteer help	7%	5%	6%	9%	8%	7%	0%	11%

Labor shortages, on the other hand, were reported by roughly 60% of respondents, and appear to be more nuanced, depending on regional labor sources. In California and Florida, greater proportions of respondents reported challenges related to immigration restrictions, while proportionally greater Minnesota-Wisconsin respondents reported on loss of volunteers with community-based logistics such as loading and unloading deliveries. In all regions, proportions of respondents experiencing labor shortages due to sickness were higher in the second half of the year, while labor shortages due to health measure compliance disappeared by July 2020.

To measure how businesses’ experiences translated into impacts, Table 4 summarizes the changes in sales revenue in 2020 from their typical experiences by quarter, supply chain segment, and study region. The number of responses in each cell are 17 to 94 for California and Minnesota-Wisconsin samples. The number of quarterly responses is 9 to 12 for Florida’s retail and restaurants, but there are fewer in the upstream segments, perhaps driven by survey fatigue as the production agriculture businesses had been surveyed previously by the University of Florida regarding impacts experienced in the March-May 2020 period.

**Table 4:** Summary of changes in sales revenue in 2020, by quarter, supply chain segment, and study region.

		Q1			Q2			Q3			Q4		
		*N*	*Mean*	(*SD*)	*N*	*Mean*	(*SD*)	*N*	*Mean*	(*SD*)	*N*	*Mean*	(*SD*)
**California**													
	Production agriculture	27	-20.37	(43.02)	37	-33.08	(52.71)	38	-26.84	(47.33)	37	-30.14	(51.15)
	Food processing/manufacturing	22	-17.36	(33.43)	23	-52.26	(44.43)	23	-34.17	(43.57)	23	-28.87	(45.51)
	Grocery wholesale	17	-16.88	(61.83)	21	-37.43	(50.41)	21	-33.14	(42.65)	23	-25.13	(42.22)
	Food and beverage retail	52	-25.08	(36.06)	62	-46.74	(42.84)	63	-39.87	(46.96)	64	-34.38	(48.43)
	Restaurants	81	-30.79	(32.20)	92	-54.68	(37.66)	92	-42.75	(34.78)	94	-40.20	(40.45)
**Florida**													
	Production agriculture	1	NA		5	50.20	(46.5)	3	27.67	(5.77)	3	-6.67	(26.56)
	Food processing/manufacturing	3	-32.33	(21.94)	3	-20.00	(8.66)	3	0.00	(17.32)	3	1.67	(5.77)
	Grocery wholesale	1	NA		2	9.50	(13.43)	1	NA		1	NA	
	Food and beverage retail	9	-13.11	(28.66)	12	-27.17	(25.62)	11	-24.09	(22.66)	11	-24.55	(37.29)
	Restaurants	10	-30	(30.06)	10	-63.10	(18.10)	10	-40.10	(10.00)	10	-21.5	(15.88)
**Minnesota-Wisconsin**													
	Production agriculture	24	-9.17	(44.50)	37	0.62	(44.14)	45	3.38	(38.96)	36	0.03	(34.73)
	Food processing/manufacturing	16	3.69	(42.05)	23	2.30	(38.44)	23	11.78	(34.57)	25	9.48	(41.63)
	Grocery wholesale	18	-3.06	(34.20)	22	-11.41	(31.22)	20	-7.35	(33.11)	23	-8.87	(36.33)
	Food and beverage retail	41	-0.76	(37.55)	45	-14.60	(44.30)	46	-9.80	(39.83)	48	-5.85	(49.20)
	Restaurants	44	-24.9	(23.07)	48	-54.73	(28.95)	48	-39.46	(34.93)	50	-46.96	(32.43)

**Fig 1:**
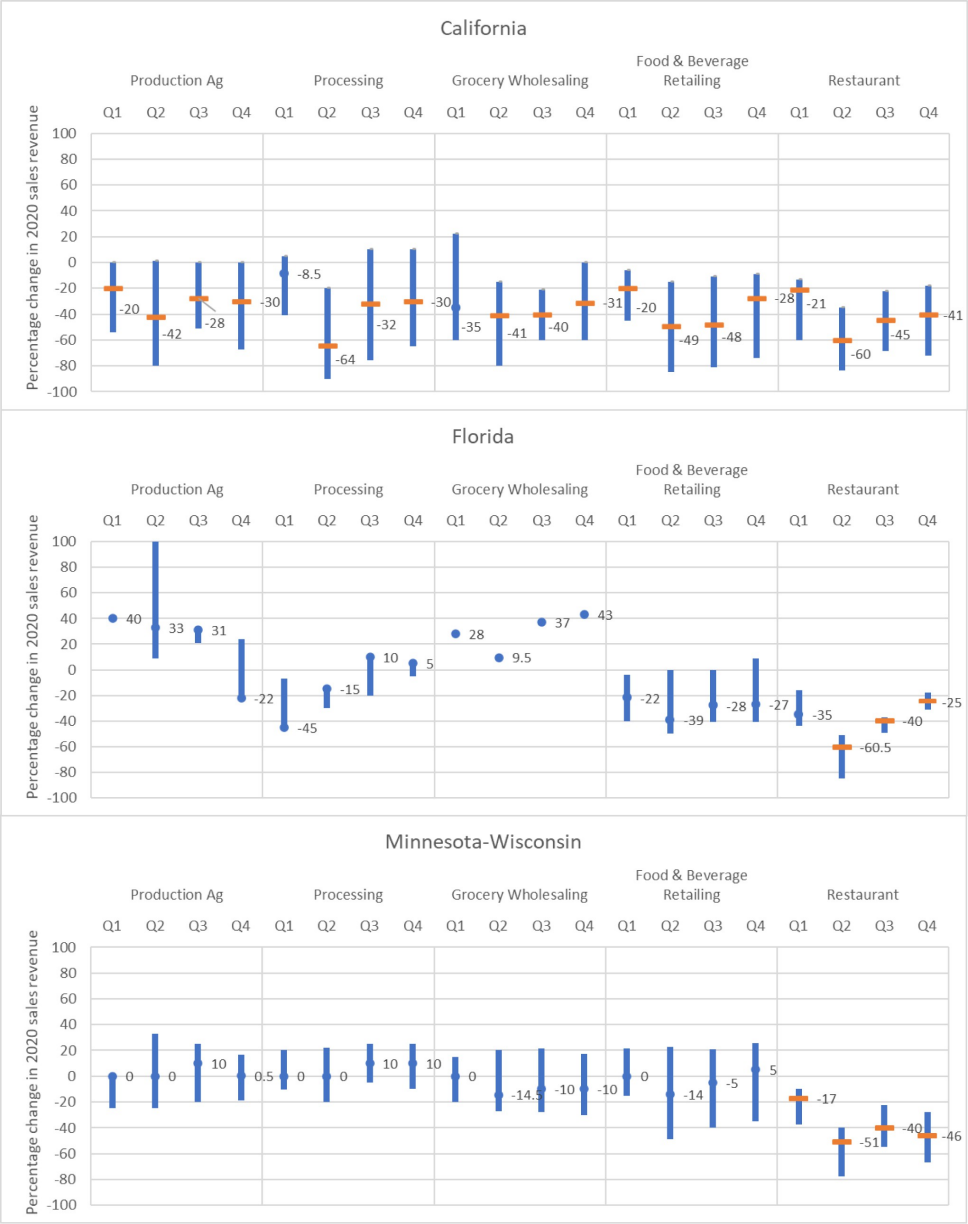
Quarterly changes to business sales revenue relative to pre-pandemic levels, 2020^a^. ^a^Bars represent 75th and 25th percentiles of responses. Numbers indicate the median value. Wide ticks represent statistical significance at the 5% level. Sample sizes are reported in Table 4.

Fig 1 depicts the 25^th^ to 75^th^ percentiles of quarterly responses for the agri-food supply chain segments in the three study regions. A wide tick mark signifies that the null hypothesis of no change in sales revenues from pre-pandemic levels is rejected by the sign test at the 5% level, meaning that the change in sales revenue reported by survey respondents is statistically significant. In California, the decline in sales revenue was statistically significant in all supply chain segments starting in the second quarter of 2020. Agricultural producers, retailers, and restaurants were already impacted with a decline in sales revenue in March. In Minnesota-Wisconsin, only the responses from the restaurants indicated statistically significant declines in sales revenue throughout the year. Although the Florida sample size is too limited to make any statistical inferences by supply chain segments, their story appears to be more like that of Minnesota-Wisconsin than California.

Pairwise comparison results (reported in a S1 Appendix) confirm that in Minnesota-Wisconsin, restaurants’ collective experience was statistically different (p-values < .005) from the other supply chain segments in all four quarters of 2020. Moreover, their experience varied during the first three quarters (p-values < .02), suggesting a volatile period for these business owners. In California, of forty pairwise tests among supply chain segments within each quarter, only four pairs were statistically different from each other at the 5% level, supporting the conclusion that the pandemic’s impact was shared widely across the supply chain segments. As in the Minnesota-Wisconsin region, California restaurants’ experience was more variable across quarters than other segments of the supply chain; five out of six pairs were statistically significant, compared to two and one for the retail and processing segments, respectively.

## Discussion

We find significant differences in the economic impact of the COVID-19 pandemic on agri-food supply chain operations across market segments and geographic regions. Of the study regions, only the experience of Minnesota-Wisconsin was similar to the generalized statements made about the US or Canadian supply chains, which appeared to have coped with the exception of restaurants (and other food service sectors) (e.g., [7, 20]). While the average magnitudes of the decline in sales are comparable to the past reported values (e.g., [5, 6, 21]), firm-level responses reveal substantial variability across businesses. Broadly, two factors likely contributed to these regional differences: (1) regional disparities in pandemic evolution and governance, and (2) structural differences in regional agri-food systems.

**Fig 2:**
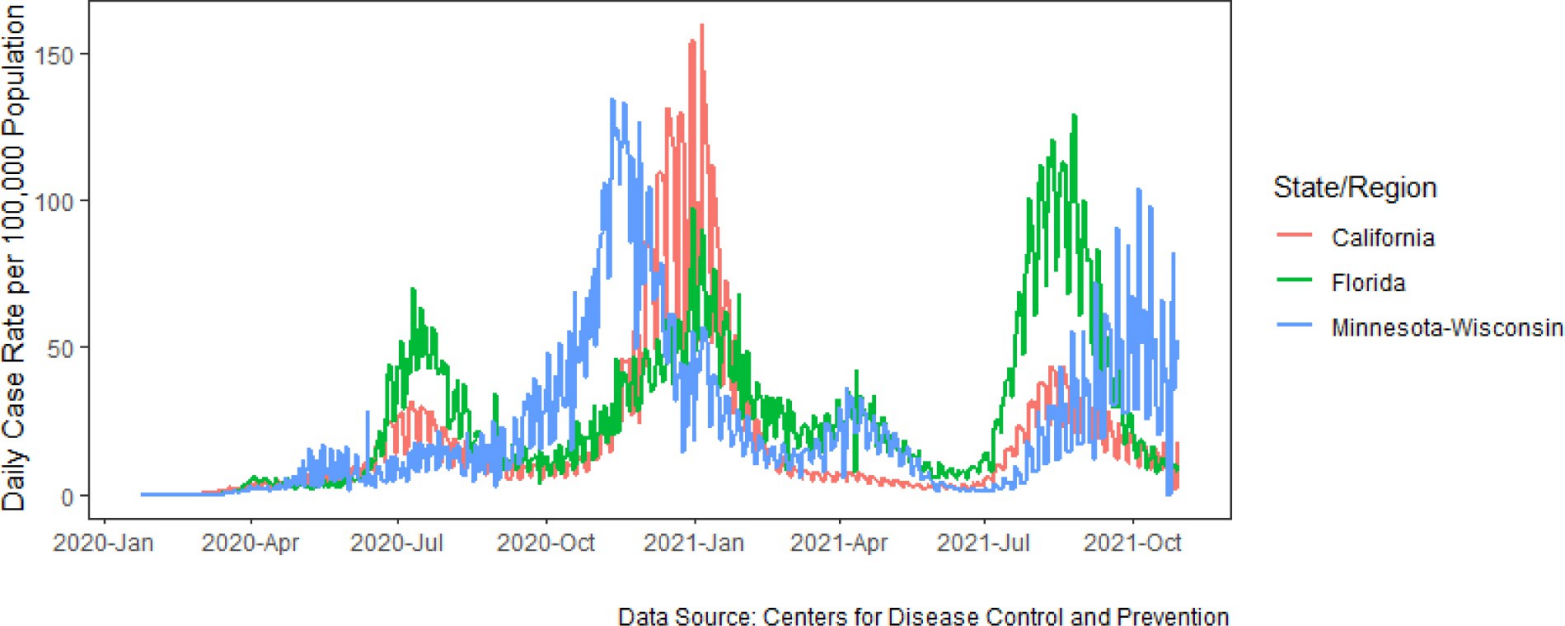
Daily case rate per 100,000 population in Florida, California, and the combined region of Minnesota-Wisconsin.

Fig 2 illustrates the trends in reported cases of COVID-19 per 100,000 population between January 2020 and November 2021. Over this period, case rates spiked at different times and reached different peaks across the three regions of interest. The impact of the pandemic on daily life, and in turn the agri-food supply chain, was also heterogeneous across these regions and was largely dependent on public health measures implemented to mitigate the spread of COVID-19. Public health measures began in all regions in March 2020, but they varied significantly in terms of restrictiveness, implementation period, and thresholds considered for businesses to reopen. While the initial surge in cases occurred in the summer of 2020 for California and Florida, it was only in the late third quarter that the cases began to surge in Minnesota-Wisconsin. This trend in cases corroborates the survey responses where respondents from California and Florida reported greater negative impacts from the pandemic throughout most of 2020. Nonetheless, measures such as limited seating capacity in restaurants and bars were implemented for relatively extended periods in all regions, affecting the segment. Throughout the pandemic, each of the four states in our study also announced varying state-level loan programs and relief packages to support local, small, and/or ethnically diverse businesses that were at risk for financial hardship due to the ongoing pandemic, in addition to these businesses being eligible for similar federal-level programs. The timing and magnitude of subsequent surges in 2021 suggest likely differential impacts on the regional agri-food systems. As cases resurged in California and Florida during the first quarter of 2021, the spread had slowed in Minnesota-Wisconsin. Another surge in the third quarter of 2021 was much greater in Florida than in California.

At the business level, COVID-19 disruption effects differ by region in part because of the seasonal nature of food production. For instance, one survey respondent stated in answer to a write-in question that “As a seasonal business [which] thrives in the winter/spring months, the timing of the closures in 2020 couldn’t have been worse for us.” This response and others like it may be explained by the fact that California’s rolling seasonal produce was not yet ready for harvest when labor markets contracted, limiting sales throughout the year. Conversely, Florida’s stability in second quarter sales revenue might reflect completed harvests and fixed sales contracts. Similarly, Minnesota-Wisconsin sales revenue for the third quarter correspond to the seasonal harvest window and might indicate that market opportunities were greater as the food flow from western states constricted due to labor shortages during key production windows, such as planting and harvest.

In addition to seasonality, the relative size and structure of food systems are likely relevant, determining how quickly and effectively businesses were able to pivot and adapt to disruptions from COVID-19. Agri-food systems of Minnesota and Wisconsin are much more balanced across the supply chain segments than those of California and Florida, where the downstream half of the segments constitute 65% and 79% of total sales revenue (Table 1). It is likely that the more unbalanced food supply chain structure perpetuated cascading systems failures [22, 23].

The tradeoff between efficiency from operation scale and resilience from diversity is discussed in the context of sustainability [24]. Large scale operations are needed for efficiency, while a set of smaller-scale, sufficiently differentiated operations can contribute to overall resilience with their abilities to innovate and build stable business relationships [25]. Similarly, the relative concentration of food systems (particularly in processing, wholesale, and distribution) increases efficiency but sacrifices resilience [26]. Further research is needed to test whether regions with more diversity in terms of smaller, more differentiated businesses along the agri-food supply chain fared relatively better. Given the significant differences in businesses’ responses to COVID-19 across supply chain segments and regions, preparedness for future pandemics, natural disasters and human-made crises will require the development of regionalized, and perhaps localized, best practices for operations of various scales that are sufficiently differentiated in terms of inputs and outputs.

## Supporting information

S1 Appendix Two-sample Wilcoxon rank-sum (Mann-Whitney) test, Prob>|z|. (PDF)

## References

[1] Tendall D. M., Joerin J., Kopainsky B., Edwards P., Shreck A., Le Q. B., et al. (2015). Food system resilience: Defining the concept. *Global Food Security*, 6, 17–23. 10.1016/j.gfs.2015.08.001.

[2] OECD (The Organisation for Economic Co-operation and Development). (2020, June 2). Food Supply Chains and COVID-19: Impacts and Policy Lessons [Online]. http://www.oecd.org/coronavirus/policy-responses/food-supply-chains-and-covid-19-impacts-and-policy-lessons-71b57aea/. Accessed on May 31, 2022.

[3] Hobbs J. E. (2021). Food supply chain resilience and the COVID-19 pandemic: What have we learned? Canadian Journal of Agricultural Economics, Special Issue. 69:189–196. doi: 10.1111/cjag.12279

[4] Peña-Lévano L., Burney S., Adams C. (2020). Labor disruptions caused by COVID-19 in the U.S. agriculture and nonfarm industries. Choices. Quarter 3. Available at: https://www.choicesmagazine.org/UserFiles/file/cmsarticle_753.pdf

[5] Fairlie R., & Fossen F. M. (2021). The early impacts of the COVID-19 pandemic on business sales. Small Business Economics, 58(4), 1853–1864. 10.1007/s11187-021-00479-4 PMC8009687 38624577

[6] Goddard E. (2021). The impact of COVID‐19 on food retail and food service in Canada: A second assessment. *Canadian Journal of Agricultural Economics/Revue canadienne d‘agroeconomie*, 69(2), 167–175.

[7] Chenarides L., Richards T. J., & Rickard B. (2021). COVID‐19 impact on fruit and vegetable markets: One year later. *Canadian Journal of Agricultural Economics/Revue canadienne d’agroeconomie*, 69(2), 203–214.

[8] Höhler J. and Lansink A.O. (2021). Measuring the impact of COVID-19 on stock prices and profits in the food supply chain. *Agribusiness*. 27:171–186. doi: 10.1002/agr.21678 33362339 PMC7753738

[9] Goodrich B., Kiesel K., Bruno E. (2021). Differential impacts of the COVID-19 pandemic on California’s produce and nut industries. Western Economics Forum. 19(1): 58–74.

[10] Hailu G. (2020). Economic thoughts on COVID‐19 for Canadian food processors. *Canadian 11*. *Journal of Agricultural Economics*. 68, 163–169. doi: 10.1111/cjag.12241

[11] Padilla S., Schulz L.L., Vaiknoras K., MacLachlan M.J. (2021). COVID-19 working paper: Changes in regional hog slaughter during COVID-19. USDA, Economic Research Service. No. AP-095.

[12] Hall C. M., Scott D., & Gössling S. (2020). Pandemics, transformations and tourism: Be careful what you wish for. Tourism Geographies, 1–22. 10.1080/14616688.2020.1759131

[13] Anderson JD, Mitchell JL, Maples JG. Invited review: Lessons from the COVID-19 pandemic for food supply chains. *Applied Animal Science*. 37: 738–747. doi: 10.15232/aas.2021-02223

[14] Rejeb A., Rejeb K., Keogh JG. (2020). COVID-19 and the food chain? Impacts and future research trends. *Scientific Journal of Logistics*. 16(4): 475–485. doi: 10.17270/J.LOG.2020.502

[15] Balagtas J. V. and Cooper J. (2021) The impact of COVID-19 on United States meat and livestock markets. *Choices Magazine*. 36(3): 1–10.

[16] Hayes D.J., Schulz L.L., Hart C.E., Jacobs K.L. (2021). A descriptive analysis of the COVID-19 impacts on US pork, turkey and egg markets. *Agribusiness*. 37: 122–141. doi: 10.1002/agr.21674 33362337 PMC7753661

[17] Lusk J. L., Tonsor G. T., & Schulz L. L. (2021). Beef and pork marketing margins and price spreads during COVID‐19. *Applied Economic Perspectives and Policy*, 43(1), 4–23. doi: 10.1002/aepp.13101 33042511 PMC7537190

[18] Malone T., Schaefer K. A., & Lusk J. L. (2021). Unscrambling US egg supply chains amid COVID-19. *Food Policy*, 101, 102046. 36570063 10.1016/j.foodpol.2021.102046 PMC9758591

[19] Court C. D., Hodges A. W., & Lollar M. (2020). Harmonizing the assessment of the impacts of natural disasters to Florida agriculture (EDIS Publication FE1075). University of Florida, UF/IFAS Extension. 10.32473/edis-fe1075-2020.

[20] Gray R. S. (2020). Agriculture, transportation, and the COVID‐19 crisis. *Canadian Journal of Agricultural Economics/Revue canadienne d’agroeconomie*, 68(2), 239–243.

[21] Bloom N, Fletcher R.S., Yeh E. (2021). The Impact of COVID-19 on US Firms. National Bureau of Economic Research, NBER Working Paper No. w28314.

[22] Hynes W., Trump B., Love P., & Linkov I. (2020). Bouncing forward: a resilience approach to dealing with COVID-19 and future systemic shocks. *Environment Systems and Decisions*, 40, 174–184. doi: 10.1007/s10669-020-09776-x 32837818 PMC7247742

[23] Swanson D. and Santamaria L. (2021). Pandemic supply chain research: A structured literature review and bibliometric network analysis. Logistics. 5(7): 1–22.

[24] Ulanowicz R. E., Goerner S. J., Lietaer B., & Gomez R. (2009). Quantifying sustainability: Resilience, efficiency and the return of information theory. Ecological complexity, 6(1), 27–36.

[25] King R.P., Hand M.S., DiGiacomo G., Clancy K., Gomez M.I., Hardesty S., et al., McLaughlin E. (2010). Comparing the structure, size and performance of local and mainstream food supply chains. USDA, Economic Research Service. Report Number 99. https://www.ers.usda.gov/webdocs/publications/46405/7029_err99_1_.pdf?v=8753.2.

[26] Hendrickson M.K. (2020). Covid lays bare the brittleness of a concentrated and consolidated food system. *Agriculture and Human Values*, 37, 579–580. doi: 10.1007/s10460-020-10092-y 32398891 PMC7214846

[27] Healthy Foods, Healthy Lives Institute. (n.d.). 2017 State Fact Sheets [Online data files]. University of Minnesota, Healthy Foods, Healthy Foods Institute. https://hfhl.umn.edu/statefoodindicators.

[28] King R.P., Anderson M., DiGiacomo G., Mulla D., & Wallinga D. (2016). State level Food System Indicators [Project Report]. University of Minnesota, Healthy Foods, Healthy Lives Institute. https://hfhl.umn.edu/statefoodindicators

[29] U.S. Census Bureau. (2020). 2017 SUSB Annual Datasets by Establishment Industry [Data set]. U.S. Census Bureau. https://www.census.gov/data/datasets/2017/econ/susb/2017-susb.html

