# Supplementary material for: Impacts of COVID-19 on US agri-food supply chain businesses: Regional survey results
Source: PLoS One. 2023 Feb 22;18(2):e0281930. doi: 10.1371/journal.pone.0281930 (PMC9946254; doi:10.1371/journal.pone.0281930)
Supplement: S1 Appendix — (PDF) [file pone.0281930.s001.pdf]

Supplemental Appendix. Two-sample Wilcoxon rank-sum (Mann-Whitney) test, Prob>|z|

Table A1. Full table

Columns: Q1

				Q1														
				CA					FL					MW				
			N	prodag	process	whsl	retail	restau	prodag	process	whsl	retail	restau	prodag	process	whsl	retail	restau
Q1	CA	prodag	27															
Q1	CA	process	22	0.519														
Q1	CA	whsl	17	0.847	0.561													
Q1	CA	retail	52	0.792	0.239	0.807												
Q1	CA	restau	81	0.237	0.036	0.929	0.259											
Q1	FL	prodag	1	0.154														
Q1	FL	process	3		0.335				0.157									
Q1	FL	whsl	1			0.386			0.317	0.157								
Q1	FL	retail	9				0.555		0.113	0.225	0.378							
Q1	FL	restau	10					0.939	0.111	0.610	0.111	0.251						
Q1	MW	prodag	24	0.136					0.192									
Q1	MW	process	16		0.109					0.058				0.397				
Q1	MW	whsl	18			0.198					0.201			0.562	0.567			
Q1	MW	retail	41				0.000					0.176		0.295	0.922	0.692		
Q1	MW	restau	44					0.098					0.337	0.003	0.000	0.003	0.000	
Q2	CA	prodag	37	0.242														
Q2	CA	process	23		0.009													
Q2	CA	whsl	21			0.355												
Q2	CA	retail	62				0.004											
Q2	CA	restau	92					0.000										
Q2	FL	prodag	5						0.763									
Q2	FL	process	3							0.500								
Q2	FL	whsl	2								0.221							
Q2	FL	retail	12									0.317						
Q2	FL	restau	10										0.014					
Q2	MW	prodag	37											0.393				
Q2	MW	process	23												0.886			
Q2	MW	whsl	22													0.462		
Q2	MW	retail	45														0.135	
Q2	MW	restau	48															0.000
Q3	CA	prodag	38	0.631														
Q3	CA	process	23		0.228													
Q3	CA	whsl	21			0.567												
Q3	CA	retail	63				0.041											
Q3	CA	restau	92					0.005										
Q3	FL	prodag	3						0.157									
Q3	FL	process	3							0.116								
Q3	FL	whsl	1								0.317							
Q3	FL	retail	11									0.422						
Q3	FL	restau	10										0.404					
Q3	MW	prodag	45											0.083				
Q3	MW	process	23												0.415			
Q3	MW	whsl	20													0.715		
Q3	MW	retail	46														0.326	
Q3	MW	restau	48															0.004
Q4	CA	prodag	37	0.380														
Q4	CA	process	23		0.317													
Q4	CA	whsl	23			0.913												
Q4	CA	retail	64				0.147											
Q4	CA	restau	94					0.031										
Q4	FL	prodag	3						0.157									
Q4	FL	process	3							0.043								
Q4	FL	whsl	1								0.317							
Q4	FL	retail	11									0.380						
Q4	FL	restau	10										0.448					
Q4	MW	prodag	36											0.219				
Q4	MW	process	25												0.477			
Q4	MW	whsl	23													0.617		
Q4	MW	retail	48														0.848	
Q4	MW	restau	50															0.000

Columns: Q2

			Q2														
			CA					FL					MW				
			prodag	process	whsl	retail	restau	prodag	process	whsl	retail	restau	prodag	process	whsl	retail	restau
Q2	CA	prodag															
Q2	CA	process	0.168														
Q2	CA	whsl	0.795	0.284													
Q2	CA	retail	0.294	0.555	0.539												
Q2	CA	restau	0.049	0.919	0.166	0.326											
Q2	FL	prodag	0.007														
Q2	FL	process		0.159				0.023									
Q2	FL	whsl			0.172			0.237	0.076								
Q2	FL	retail				0.100		0.006	0.468	0.097							
Q2	FL	restau					0.673	0.002	0.014	0.031	0.001						
Q2	MW	prodag	0.003					0.062									
Q2	MW	process		0.000					0.184				0.970				
Q2	MW	whsl			0.033					0.464			0.252	0.211			
Q2	MW	retail				0.001					0.272		0.166	0.202	0.963		
Q2	MW	restau					0.624					0.224	0.000	0.000	0.000	0.000	
Q3	CA	prodag	0.444														
Q3	CA	process		0.140													
Q3	CA	whsl			0.571												
Q3	CA	retail				0.499											
Q3	CA	restau					0.009										
Q3	FL	prodag						0.649									
Q3	FL	process							0.261								
Q3	FL	whsl								0.221							
Q3	FL	retail									0.710						
Q3	FL	restau										0.002					
Q3	MW	prodag											0.678				
Q3	MW	process												0.306			
Q3	MW	whsl													0.677		
Q3	MW	retail														0.664	
Q3	MW	restau															0.011
Q4	CA	prodag	0.762														
Q4	CA	process		0.088													
Q4	CA	whsl			0.204												
Q4	CA	retail				0.175											
Q4	CA	restau					0.007										
Q4	FL	prodag						0.095									
Q4	FL	process							0.043								
Q4	FL	whsl								0.221							
Q4	FL	retail									0.758						
Q4	FL	restau										0.001					
Q4	MW	prodag											0.960				
Q4	MW	process												0.397			
Q4	MW	whsl													0.785		
Q4	MW	retail														0.372	
Q4	MW	restau															0.207

Columns: Q3

			Q3														
			CA					FL					MW				
			prodag	process	whsl	retail	restau	prodag	process	whsl	retail	restau	prodag	process	whsl	retail	restau
Q3	CA	prodag															
Q3	CA	process	0.062														
Q3	CA	whsl	0.375	0.963													
Q3	CA	retail	0.222	0.564	0.580												
Q3	CA	restau	0.054	0.376	0.481	0.888											
Q3	FL	prodag	0.031														
Q3	FL	process		0.212				0.043									
Q3	FL	whsl			0.134			0.157	0.157								
Q3	FL	retail				0.169		0.010	0.050	0.108							
Q3	FL	restau					0.558	0.011	0.014	0.111	0.177						
Q3	MW	prodag	0.002					0.147									
Q3	MW	process		0.002					0.600				0.604				
Q3	MW	whsl			0.013					0.137			0.208	0.102			
Q3	MW	retail				0.001					0.183		0.120	0.053	0.878		
Q3	MW	restau					0.466					0.711	0.000	0.000	0.001	0.000	
Q4	CA	prodag	0.754														
Q4	CA	process		0.869													
Q4	CA	whsl			0.459												
Q4	CA	retail				0.510											
Q4	CA	restau					0.718										
Q4	FL	prodag						0.116									
Q4	FL	process							0.500								
Q4	FL	whsl								0.317							
Q4	FL	retail									0.668						
Q4	FL	restau										0.002					
Q4	MW	prodag											0.481				
Q4	MW	process												0.959			
Q4	MW	whsl													0.913		
Q4	MW	retail														0.628	
Q4	MW	restau															0.260

Columns: Q4

			Q4														
			CA					FL					MW				
			prodag	process	whsl	retail	restau	prodag	process	whsl	retail	restau	prodag	process	whsl	retail	restau
Q4	CA	prodag															
Q4	CA	process	0.843														
Q4	CA	whsl	0.704	0.660													
Q4	CA	retail	0.775	0.630	0.358												
Q4	CA	restau	0.396	0.283	0.143	0.520											
Q4	FL	prodag	0.425														
Q4	FL	process		0.244				0.500									
Q4	FL	whsl			0.129			0.157	0.157								
Q4	FL	retail				0.491		0.238	0.481	0.108							
Q4	FL	restau					0.057	0.234	0.041	0.111	0.525						
Q4	MW	prodag	0.003					0.561									
Q4	MW	process		0.004					0.503				0.343				
Q4	MW	whsl			0.079					0.129			0.347	0.081			
Q4	MW	retail				0.002					0.134		0.724	0.306	0.597		
Q4	MW	restau					0.431					0.007	0.000	0.000	0.000	0.000	

Table A2a. Upper left quadrant

				Q1															Q2														
				CA					FL					MW					CA					FL					MW				
			N	prodag	process	whsl	retail	restau	prodag	process	whsl	retail	restau	prodag	process	whsl	retail	restau	prodag	process	whsl	retail	restau	prodag	process	whsl	retail	restau	prodag	process	whsl	retail	restau
Q1	CA	prodag	27																														
		process	22	0.519																													
		whsl	17	0.847	0.561																												
		retail	52	0.792	0.239	0.807																											
		restau	81	0.237	0.036	0.929	0.259																										
	FL	prodag	1	0.154																													
		process	3		0.335				0.157																								
		whsl	1			0.386			0.317	0.157																							
		retail	9				0.555		0.113	0.225	0.378																						
		restau	10					0.939	0.111	0.610	0.111	0.251																					
	MW	prodag	24	0.136					0.192																								
		process	16		0.109					0.058				0.397																			
		whsl	18			0.198					0.201			0.562	0.567																		
		retail	41				0.000					0.176		0.295	0.922	0.692																	
		restau	44					0.098					0.337	0.003	0.000	0.003	0.000																
Q2	CA	prodag	37	0.242																													
		process	23		0.009														0.168														
		whsl	21			0.355													0.795	0.284													
		retail	62				0.004												0.294	0.555	0.539												
		restau	92					0.000											0.049	0.919	0.166	0.326											
	FL	prodag	5						0.763										0.007														
		process	3							0.500										0.159				0.023									
		whsl	2								0.221										0.172			0.237	0.076								
		retail	12									0.317										0.100		0.006	0.468	0.097							
		restau	10										0.014										0.673	0.002	0.014	0.031	0.001						
	MW	prodag	37											0.393					0.003					0.062									
		process	23												0.886					0.000					0.184				0.970				
		whsl	22													0.462					0.033					0.464			0.252	0.211			
		retail	45														0.135					0.001					0.272		0.166	0.202	0.963		
		restau	48															0.000					0.624					0.224	0.000	0.000	0.000	0.000	

Table A2b. Bottom left quadrant

				Q1															Q2														
				CA					FL					MW					CA					FL					MW				
			N	prodag	process	whsl	retail	restau	prodag	process	whsl	retail	restau	prodag	process	whsl	retail	restau	prodag	process	whsl	retail	restau	prodag	process	whsl	retail	restau	prodag	process	whsl	retail	restau
		prodag	38	0.631															0.444														
		process	23		0.228															0.140													
	CA	whsl	21			0.567															0.571												
		retail	63				0.041															0.499											
		restau	92					0.005															0.009										
		prodag	3						0.157															0.649									
		process	3							0.116															0.261								
Q3	FL	whsl	1								0.317															0.221							
		retail	11									0.422															0.710						
		restau	10										0.404															0.002					
		prodag	45											0.083															0.678				
		process	23												0.415															0.306			
	MW	whsl	20													0.715															0.677		
		retail	46														0.326															0.664	
		restau	48															0.004															0.011
		prodag	37	0.380															0.762														
		process	23		0.317															0.088													
	CA	whsl	23			0.913															0.204												
		retail	64				0.147															0.175											
		restau	94					0.031															0.007										
		prodag	3						0.157															0.095									
		process	3							0.043															0.043								
Q4	FL	whsl	1								0.317															0.221							
		retail	11									0.380															0.758						
		restau	10										0.448															0.001					
		prodag	36											0.219															0.960				
		process	25												0.477															0.397			
	MW	whsl	23													0.617															0.785		
		retail	48														0.848															0.372	
		restau	50															0.000															0.207

Table A2c. Bottom right quadrant

				Q3															Q4														
				CA					FL					MW					CA					FL					MW				
			N	prodag	process	whsl	retail	restau	prodag	process	whsl	retail	restau	prodag	process	whsl	retail	restau	prodag	process	whsl	retail	restau	prodag	process	whsl	retail	restau	prodag	process	whsl	retail	restau
Q3	CA	prodag	38																														
		process	23	0.062																													
		whsl	21	0.375	0.963																												
		retail	63	0.222	0.564	0.580																											
		restau	92	0.054	0.376	0.481	0.888																										
	FL	prodag	3	0.031																													
		process	3		0.212				0.043																								
		whsl	1			0.134			0.157	0.157																							
		retail	11				0.169		0.010	0.050	0.108																						
		restau	10					0.558	0.011	0.014	0.111	0.177																					
	MW	prodag	45	0.002					0.147																								
		process	23		0.002					0.600				0.604																			
		whsl	20			0.013					0.137			0.208	0.102																		
		retail	46				0.001					0.183		0.120	0.053	0.878																	
		restau	48					0.466					0.711	0.000	0.000	0.001	0.000																
Q4	CA	prodag	37	0.754																													
		process	23		0.869														0.843														
		whsl	23			0.459													0.704	0.660													
		retail	64				0.510												0.775	0.630	0.358												
		restau	94					0.718											0.396	0.283	0.143	0.520											
	FL	prodag	3						0.116										0.425														
		process	3							0.500										0.244				0.500									
		whsl	1								0.317										0.129			0.157	0.157								
		retail	11									0.668										0.491		0.238	0.481	0.108							
		restau	10										0.002										0.057	0.234	0.041	0.111	0.525						
	MW	prodag	36											0.481					0.003					0.561									
		process	25												0.959					0.004					0.503				0.343				
		whsl	23													0.913					0.079					0.129			0.347	0.081			
		retail	48														0.628					0.002					0.134		0.724	0.306	0.597		
		restau	50															0.260					0.431					0.007	0.000	0.000	0.000	0.000	
